# Decreased Vitamin B_12_ Levels in Children with Nocturnal Enuresis

**DOI:** 10.5402/2012/789706

**Published:** 2012-01-26

**Authors:** Bülent Altunoluk, Mehmet Davutoglu, Mesut Garipardic, Vedat Bakan

**Affiliations:** ^1^Department of Urology, Faculty of Medicine, Kahramanmaras Sutcu Imam University, Yörükselim Mahallesi Hastane Caddesi 32, 46100 Kahramanmaras, Turkey; ^2^Department of Pediatrics, Faculty of Medicine, Kahramanmaras Sutcu Imam University, 46100 Kahramanmaras, Turkey; ^3^Department of Pediatric Surgery, Faculty of Medicine, Kahramanmaras Sutcu Imam University, 46100 Kahramanmaras, Turkey

## Abstract

*Objectives*. Nocturnal enuresis is a common pediatric problem, the etiology of which is unclear. In the present study, vitamin B_12_ and folate levels were measured in children with nocturnal enuresis and compared with those in healthy control group children to investigate whether there was any relation with enuresis and neurogenic maturation as a first time in the literature. *Methods*. In this cross-sectional study, we included thirty children (16 girls, 14 boys) who had presented with primary nocturnal enuresis (PNE) complaints in the study group and 31 children (13 girls, 18 boys) in the control group. Body weight and height measurements were obtained and complete blood counts and vitamin B_12_ and folate levels were measured in all children. *Results*. No difference was found in age, height, and weight between study and control groups. Also the mean levels of the hemoglobin, hematocrit, and mean corpuscular volume (MCV) were not different between the two groups. Significantly lower mean vitamin B_12_ and folate levels were found in the enuresis group compared with the control group. *Conclusions*. Further studies are needed to clarify B_12_ and folate deficiency in larger series so that these tests can be included in routine investigations of enuretic children.

## 1. Introduction

Primary nocturnal enuresis (PNE) is defined as involuntary voiding during the night in children after the age at which bladder control would normally be expected and is the most common voiding problem in pediatric population [1]. Because of its high prevalence, nocturnal enuresis has remained a focus of extensive scientific research over the past few decades. The etiology of PNE has been widely debated but is still not completely understood. The most commonly established causes of nocturnal enuresis are small bladder size, abnormal sleep patterns, the high amount of urine produced during sleep at night, and delayed functional maturation of the central nervous system [2, 3].

Vitamin B_12_ and folate play an important role in the metabolism, development, and maturation of the nervous system, even though their exact roles in the normal and pathologic conditions are not fully understood. In spite of extensive researches on PNE, many questions still remain unanswered regarding its exact pathophysiology. According to the best of our knowledge, there is no study that investigated serum vitamin B_12_ and folate levels in children with PNE in the English literature. 

In the present study, vitamin B_12_ and folate levels were measured in children with nocturnal enuresis and compared with those in healthy control group children to investigate whether there was any relation with enuresis and neurogenic maturation as a first time in the literature.

## 2. Material and Methods

In this study, the study group comprised of prospectively recruited 30 children with nocturnal enuresis. Primer nocturnal enuresis was defined as bed-wetting in a child who never had a dry period for over 6 months according to the International Children Continence Society recommendations [4]. Enuretic children were selected among new consecutive referrals to our outpatient clinics meeting the following inclusion criteria: a frequency of two or more enuretic episodes per week, no associated daytime wetting, no dry period of more than 3-month duration since birth, normal pubertal stage for age assessed by the Tanner staging [5], no neurological or urological abnormalities, normal blood biochemical analysis, normal urine analysis, including urinary culture, no history of previous urinary tract infection, and no other signs or symptoms of any chronic illness. Thirty-one matched healthy children with similar age and without enuresis were included as the control group. Children having a current or prior history of nervous system diseases were not included.

Body weight and height measurements of the children were obtained carefully by the same experienced personnel. Complete blood counts and vitamin B_12_ and folate levels were measured in all children. The cutoff points used for serum folic acid were <3 ng/ml for deficiency, 3–6 ng/mL for low levels, and >6 ng/mL for normal values. Vitamin B_12_ deficiency was defined as serum concentration below 200 pg/mL, normal range 200 to 900 pg/mL, and excess >900 pg/mL.

Institutional Local Ethics Committee approved the study protocol, and written informed consent was obtained from the parents of the children after explaining the nature of the procedures. The results were expressed as mean ± standard deviation (SD) and the range. The paired samples *t*-test and Mann-Whitney *U* test were used for statistical analyses. A *P* value less than 0.05 was considered statistically significant.

## 3. Results

Thirty children (16 girls, 14 boys) in the study group and 31 children (13 girls, 18 boys) in the control group were enrolled in the study. The mean age of the children in the enuresis group was 9.3 ± 2.7 years (range 5–14 years) and 9.52 ± 1.9 years (range 5–14 years) in the control group. Of 61 subjects included in the study, no difference was found in age, height, and weight between study and control groups (*P* > 0.05; Table 1). In addition, the mean levels of the hemoglobin, hematocrit, and mean corpuscular volume (MCV) were not different between the two groups (*P* > 0.05; Table 1). The parental history of enuresis was seen in 22 of 30 children in the enuresis group. On the other hand, in the control group there was no parental history of nocturnal enuresis (*P* > 0.05) (Table 1). Significantly lower mean vitamin B_12_ and folate levels were found in the enuresis group compared with the control group (*P* = 0.031 and *P* = 0.025, resp.  Table 2). Also, the number of children with vitamin B_12_ deficiency in the enuretic group was significantly higher than that in the control group (*n* = 9 (30%) versus *n* = 0 (0%), *P* = 0.003). With regard to folate deficiency, there was no folate deficiency in the control group, while there were two (6.7%) children with low serum folic acid levels in the enuretic group (Table 2).

## 4. Discussion

In the present study children with primary nocturnal enuresis tended to have lower vitamin B_12_ and folate levels than those of the control children. We searched medical indexes about serum vitamin B_12_ and folate levels in enuresis and found no study dealing with this topic. Therefore this is the first study that investigated B_12_ and folate levels in enuretic children.

Nocturnal enuresis is the repeated, involuntary loss of urine during sleep [6]. Bed-wetting spontaneously ceases with increasing age, being present in 15% of 5-year-old children, 5% of 10-year-old children, and 1% of 15-year-old children. Despite extensive research, there is still significant controversy regarding its etiology, and it is now generally accepted that multiple pathologic factors are probably involved.

Theories reported as the probable causes of enuresis delayed central nervous system maturation, psychogenic and behavioral components, environmental influences, deep sleep, allergies, small bladder capacity, uninhibited neurogenic bladder contractions, structural abnormalities of the urinary tract, lack of diurnal variation of vasopressin, nocturnal hypercalciuria, prostaglandin production, and sleep apnea [7–10]. 

Although nocturnal enuresis is one of the common problems among children, the pathophysiology of this disorder remains unclear. The most commonly emphasized pathophysiologic theory of nocturnal enuresis proposes a delayed functional maturation of the central nervous system control on the bladder at night. Factors that support this delayed maturation theory include a significant spontaneous cure rate with the increasing of age, the results of urodynamic assessments demonstrating a reduced functional bladder capacity, association of delayed motor development, a proposed deficiency in endocrine maturation, and the documented hereditary aspect of enuresis [2, 11, 12]. Additionally, retardation in the growth rate, decreased bone age and bone mineral density determined in children with nocturnal enuresis may reflect delayed maturation of regulatory central nervous system functions [3, 13].

Although there is as yet no direct neuroanatomical evidence for this hypothesis it is supported by the decrease in enuresis with advancing age so that fifteen percent of these children become dry at night each year, attesting to its developmental nature [6, 14]. 

Because of the unclear etiopathogenesis, we measured the levels of vitamin B_12_ and folate, effective on the neurogenic maturation, in enuretic children. Vitamin B_12_ and folate levels were found significantly lower in the enuresis group compared with the control group. Neurologic disease due to a lack of vitamin B_12_ is a clinical entity. Deficiency of vitamin B_12_ affects particularly hematopoietic, epithelial, and nervous tissues but the exact role of vitamin B_12_ in the metabolism of the nervous system remains unclear [15]. Deficiency of vitamin B_12_ affects mainly the central nervous system and tissues with fast mitotic activity, such as hematopoietic and digestive tract epithelium [16, 17]. The main systems affected due to B_12_ deficiency are the hematological, skin, and mucous membranes and the nervous system. Neurological features are attributable to pathology in the peripheral nerves, optic nerves, and posterior and lateral columns of the spinal cord and brain. B_12_ deficiency causes a wide spectrum of neurological manifestations ranging from neural tube defects to changes in cognition and behavior [18–20].

It is not known how cobalamin deficiency causes neurologic problems. During a deficiency state of B_12_, methylmalonyl-CoA accumulates and is used instead of acetyl-CoA in the synthesis of fatty acids. This results in unstable myelin that degrades more easily and has damaging effects on neurological development of growing children [21].

Folates are vitamins essential to the development of the central nervous system. Insufficient folate activity at the time of conception and early pregnancy can result in congenital neural tube defects. Folate is particularly important during the early development of the brain. Folate deficiency can cause megaloblastic anaemia, congestive heart failure, pigmentation, premature greying of hair, infertility, cervical dysplasia, uterine dysplasia, neuropathy, psychiatric disorders, cognitive dysfunction, and dementia. Behavioural abnormalities have been noted in folate-deficient mice [22]. 

The family history is one key because if one parent wets the bed, 44% of his or her children will do so and if both parents wet the bed, 77% of their children will do so [23].

PNE is believed by many investigators to be due to a maturational delay of the central nervous system connections necessary for nocturnal bladder control [13].

A limitation of our study is the small sample size. Further prospective studies with larger series are needed.

In conclusion, if B_12_ and folate deficiency will be detected in larger series, these tests can be included in routine investigations of enuretic children. Hence, in those children with low levels, the efficacy of the supplementation treatment may be investigated.

## Figures and Tables

**Table 1:** Comparison of the primary nocturnal enuresis (PNE) group and the control group (mean ± standard deviation).

	PNE group (*n* = 30)	Control group (*n* = 31)	*P* value
Age (year)	9.27 ± 2.66	9.52 ± 1.93	0.677
Height (cm)	125.92 ± 16.87	131.22 ± 12.92	0.173
Weight (kg)	29.53 ± 10.80	29.19 ± 9.97	0.899
Hb (gr/dl)	13.19 ± 0.73	12.96 ± 1.04	0.321
Hematocrit (%)	39.36 ± 2.12	38.23 ± 2.96	0.093
MCV(fL)	80.93 ± 4.31	78.72 ± 7.00	0.145
Parental history of enuresis (%)	22/30 73.3%	0/31 0%	<0.001

NS: not significant (*P* > 0.05).

**Table 2:** The comparison of mean vitamin B_12_ and folate levels and the percent of children having low folate and vitamin B_12_ values between the enuretic (PNE) and the control groups.

	PNE group (*n* = 30)	Control group (*n* = 31)	*P* value
Mean B_12_ level (pg/mL)	276.1 ± 93.3	343.6 ± 141	0.031
Low B_12_ (*n*, %)	9 (30%)	0 (0%)	0.003
Mean Folate level (ng/mL)	8.52 ± 1.89	9.60 ± 1.80	0.025
Low folate (*n*, %)	2 (6.7%)	0 (0%)	0.458
